# Effect of Brief Biofeedback via a Smartphone App on Stress Recovery: Randomized Experimental Study

**DOI:** 10.2196/15974

**Published:** 2019-11-26

**Authors:** John F Hunter, Meryl S Olah, Allison L Williams, Acacia C Parks, Sarah D Pressman

**Affiliations:** 1 Department of Psychological Science University of California, Irvine Irvine, CA United States; 2 Happify Health New York, NY United States

**Keywords:** heart rate variability biofeedback, stress recovery, salivary alpha amylase, smartphone, mHealth

## Abstract

**Background:**

Smartphones are often vilified for negatively influencing well-being and contributing to stress. However, these devices may, in fact, be useful in times of stress and, in particular, aid in stress recovery. Mobile apps that deliver evidence-based techniques for stress reduction, such as heart rate variability biofeedback (HRVB) training, hold promise as convenient, accessible, and effective stress-reducing tools. Numerous mobile health apps that may potentially aid in stress recovery are available, but very few have demonstrated that they can influence health-related physiological stress parameters (eg, salivary biomarkers of stress). The ability to recover swiftly from stress and reduce physiological arousal is particularly important for long-term health, and thus, it is imperative that evidence is provided to demonstrate the effectiveness of stress-reducing mobile health apps in this context.

**Objective:**

The purpose of this research was to investigate the physiological and psychological effects of using a smartphone app for HRVB training following a stressful experience. The efficacy of the gamified Breather component of the Happify mobile health app was examined in an experimental setting.

**Methods:**

In this study, participants (N=140) underwent a laboratory stressor and were randomly assigned to recover in one of three ways: with no phone present, with a phone present, with the HRBV game. Those in the *no phone* condition had no access to their phone. Those in the *phone present* condition had their phone but did not use it. Those in the *HRVB game* condition used the serious game Breather on the Happify app. Stress recovery was assessed via repeated measures of salivary alpha amylase, cortisol, and self-reported acute stress (on a 1-100 scale).

**Results:**

Participants in the *HRVB game* condition had significantly lower levels of salivary alpha amylase during recovery than participants in the other conditions (F_2,133_=3.78, *P*=.03). There were no significant differences among the conditions during recovery for salivary cortisol levels or self-reported stress.

**Conclusions:**

These results show that engaging in a brief HRVB training session on a smartphone reduces levels of salivary alpha amylase following a stressful experience, providing preliminary evidence for the effectiveness of Breather in improving physiological stress recovery. Given the known ties between stress recovery and future well-being, this study provides a possible mechanism by which gamified biofeedback apps may lead to better health.

## Introduction

### Background

Although smartphones are often criticized for contributing to ill-being [1-4], these devices hold great potential for improving one’s well-being if utilized properly in specific contexts. Smartphones may be particularly useful as tools that provide gamified apps to deliver stress-buffering interventions. Stress is prevalent in many peoples’ lives, and its accumulated effects can lead to various undesirable physical and mental health outcomes, such as an increased risk for mortality [5]. Many of these negative outcomes are due to prolonged activation of one’s stress systems [6]. However, if individuals employ strategies that promote more efficient recovery from stressors, some of the negative long-term impacts may be mitigated.

Why do smartphones present a promising opportunity for altering stress recovery? Psychologists have developed a variety of evidence-backed strategies that aid in stress reduction [7], such as biofeedback training, which directs individuals to monitor and attempt to alter their physiological arousal pattern [8]. Smartphones are ideally situated to be used as a tool for biofeedback training and to combat the negative effects of stress because they are popular, conveniently accessible, and have an array of technological capabilities [9]. Since these devices are nearly omnipresent in daily life, they can deliver interventions and assistance wherever and whenever needed.

Smartphones provide a range of possibilities for helping individuals recover from a stressful experience and may even do so when not actively used. Even when merely present, smartphones serve as symbols that can cause cognitive distraction [10] or activate representations of social connections [11]. Distraction induced by smartphones has generally been viewed as detrimental [12]; however, such distraction can be beneficial when faced with a stressor, because it can draw attention away from the negative stimuli at hand and help circumvent rumination [13]. In addition, symbolic representations of social connections can elicit perceptions of social support [14], which, when perceived passively, is the most effective form of support for stress alleviation [15]. By providing distraction and perceived social support, the mere presence of a smartphone may aid in stress recovery by serving as a “digital security blanket” in instances of social stress [16]. Thus, it is important to investigate more fully how merely having a smartphone in one’s presence may aid in stress alleviation.

Research has demonstrated that *actually using* one’s smartphone can be beneficial or detrimental for stress recovery, depending on how and when the device is used. For example, research has shown that using social media sites such as Facebook can provide social resources that sometimes help buffer acute stress [17], but at other times, fail to do so [18]. In some instances, social support gleaned via text message can reduce cardiovascular responses to stress [19]. However, sending and receiving text messages can also increase physiological indicators of stress such as heart rate, respiration, and skin conductance [20]. These mixed findings concerning how phone use influences stress underscore the fact that the ways in which we commonly interact with our devices are not universally beneficial for stress recovery. In fact, when looked at more broadly, greater use of smartphones is associated with higher levels of physiological stress [21]. Therefore, if we hope to highlight the most effective ways to use a phone to reduce stress, it may be important to go beyond natural phone use habits and, instead, provide structured apps that are specifically designed for stress reduction.

One promising way to use a smartphone to aid in stress reduction is by engaging with a mobile health (mHealth) app. mHealth apps can utilize technological capabilities (eg, phone sensors, interactive displays) and draw on the ubiquity of smartphones in everyday life to deliver functional and convenient interventions [22]. By combining evidence-based stress-reduction techniques with an engaging and ever-present medium, mHealth apps hold great promise for mitigating the negative effects of stress.

Happify is an mHealth app that provides gamified activities aimed at improving well-being and reducing stress [23]. Happify is representative of multiple aspects of other mHealth apps because it employs various smartphone technological capabilities (eg, sensors, visual and audio components, engaging interface) and incorporates empirically validated strategies to deliver training in a self-contained package. Within the Happify suite of activities, the Breather function delivers heart rate variability biofeedback (HRVB) training (Multimedia Appendix 1). HRVB is a particularly effective stress-reducing activity that targets changes in heart rate variability (HRV) by regulating breathing and bringing awareness to physiological function [24]. HRV is an index of beat-to-beat changes in heart rate and is an indicator of parasympathetic nervous system activity [25]. When undergoing a stressor, the typical response is for our sympathetic nervous system to activate and parasympathetic activity to decline (indicating low HRV). However, an adaptive response to a stressor would be for an individual to exhibit higher HRV. This is because greater fluctuations in heart rhythm (higher HRV) indicate greater adaptability to physiological needs than fewer fluctuations (lower HRV) [24]. When HRV is higher, it is a sign that our cardiovascular system (and multiple associated systems) is responding appropriately to environmental demands (eg, a stressor). Thus, using HRVB to increase HRV may be helpful when recovering from a stressor because it activates our parasympathetic nervous system and allows us to more quickly reduce physiological arousal. It is also important to note that high HRV is considered a protective factor against cardiovascular disease and is generally associated with good health and well-being [26]. Additionally, low HRV has numerous negative implications for long-term health outcomes, such as increased risk for mortality and morbidity [27,28]. HRVB training has been successfully utilized in a variety of acute stress settings and is well-validated technique for reducing stress [29,30]. The goal of undergoing a 5-minute guided session on Breather is for the user to increase HRV and recover effectively from a stressful experience.

Delivering HRVB through a smartphone app provides many advantages over traditional training. Breather overcomes barriers of nondigital HRVB interventions (eg, bulky and expensive equipment, lengthy sessions) because it is quick to administer, is portable and readily accessible, and has all the hardware and software integrated into a single device. Breather has taken advantage of mobile technology affordances to package an HRVB product in ways that should allow it to be used across a variety of stressful contexts.

**Figure 1:**
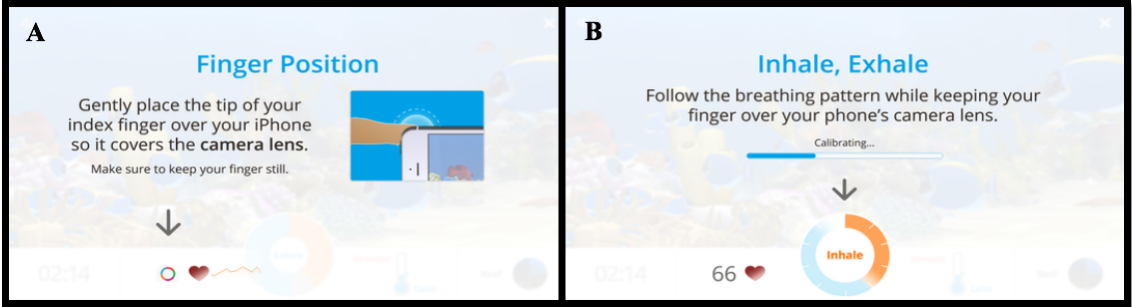
On-screen instructions for obtaining heart rate variability measurement and calibrating breathing guidance.

Users of Breather generate HRV observations by placing their index finger over the camera of their smartphone (Figure 1A). The light from the camera can be used to monitor blood volume changes within the finger. This process (ie, photoplethysmography) relies on measuring changes in light absorption on the skin of the finger. Algorithms programmed by Happify software engineers then transform those data into a simple signal that is visible to the user. The accuracy of this technology for determining HRV has been recently validated in a series of experiments that compared simultaneously obtained HRV metrics from Happify Breather and traditional electrocardiogram techniques using electrodes [31].

After calibrating the heart rate of the individual, a circular meter directs the individual to follow the breathing patterns on screen (Figure 1B). The meter directs the individual to breathe in for 4 seconds and then breathe out for 6 seconds. This 10-second breathing cycle is ideal for creating a resonant frequency (ie, breathing and heart rate align) that should maximize HRV [32].

**Figure 2:**
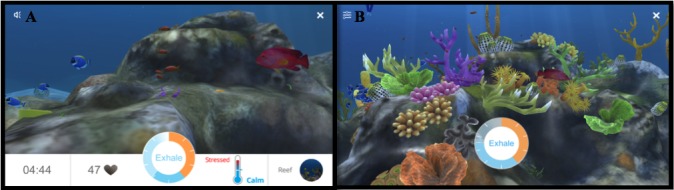
Example of how the full display unfolds when an individual is using Breather.

After calibration is complete, the interface changes into a calming nature scene (eg, underwater coral bed, tropical beach, mountaintops). The user then travels through the natural environment while he/she continues to breathe along with the meter (Figure 2A). As they breathe deeply and regularly, their HRV increases and the scene becomes more complex and beautiful (eg, coral polyps bloom, flowers grow; Figure 2B). By visually monitoring the changes in the scene, individuals are undergoing HRVB; this process is analogous to how individuals monitor electrocardiogram signals in more traditional “nongamified” HRVB trainings. The app is designed to increase HRV and reduce stress if the users adhere to the directions properly for a 5-minute session.

### Assessment of Stress

In this paper, we used a multimethodological approach to determine the effects of smartphones on stress recovery. Stress can be assessed in a variety of ways, and each method provides unique insight into the complex dynamics of how stress impacts our bodies and brains. One of the most common ways to assess stress is to ask individuals to subjectively rate their stress level. Although self-report is advantageous for assessing perceived stress, there are problems of bias (eg, self-presentation concerns) that limit the accuracy and generalizability of these assessments [33]. Due to its complexity, the most appropriate and comprehensive manner in which stress is assessed is a multimodal approach that combines subjective and objective assessments [34].

One of the most effective, reliable, and efficient ways to capture physiological measurements of stress is to analyze salivary biomarkers. Salivary cortisol is a downstream output of hypothalamic-pituitary-adrenal axis system activation and is one of the most widely used and reliable measures of physiological stress [35]. Higher levels of salivary cortisol indicate greater physiological stress. Another emerging indicator of physiological stress is salivary alpha amylase (sAA), which is an indicator of autonomic nervous system activity and is most strongly tied with sympathetic nervous system activity, the system responsible for the “fight-or-flight” response [36]. When physiologically aroused, sAA is released via the salivary glands and indicates an immediate stress response. In some cases, sAA is more strongly tied to stress and anxiety than cortisol [37,38], and sAA (but not cortisol) has also been shown to be influenced by smartphones while recovering from a stressor [16]. Thus, sAA is considered our primary outcome of interest. These salivary assessments of hypothalamic-pituitary-adrenal and autonomic nervous system activities combined with self-report give researchers a comprehensive understanding of physiological responses to stress.

### This Study

This study investigated the effectiveness of using an HRVB smartphone app to aid in stress recovery. In order to account for the potential stress-buffering effects of simply having a phone [16], we included a condition in which individuals had a phone in their presence. Thus, use of the HRVB app was compared to two control conditions, one in which no phone was present and one in which individuals had their smartphones present when recovering from a stressor.

To examine how these different types of smartphone interactions influence stress recovery, a laboratory experiment was conducted in which participants underwent a standardized stressor, used their phone in a particular way depending on their assigned condition, and were assessed on a range of psychological and physiological stress indicators. We hypothesized that those in the *HRVB game* condition would recover from the stressor more effectively than those who had their phones present or had no phone at all.

This study is one of the first empirical investigations to assess the effects of smartphone app usage on salivary biomarkers of physiological stress [39]. To our knowledge, it is also the first to examine the stress-buffering effects of an HRVB intervention delivered via a smartphone without any external equipment. In addition, since simply having a phone in your presence has been shown to aid in stress recovery, the inclusion of separate experimental conditions for a HRVB game and mere phone presence enabled us to differentiate their effects on stress. The results of this study will help our understanding of why smartphones might be helpful in times of stress, which may inform future recommendations about the most effective way to use a smartphone following a stressful experience.

## Methods

### Participants

The study was approved by the University of California, Irvine Institutional Review Board, and participants were recruited via the University of California, Irvine undergraduate psychology subject pool. These data were drawn from a larger project that included additional research questions outside the scope of this study. For this particular study, a total of 140 participants were examined (mean age 20.28, SD 2.68; 77.1% female; 45.7% Asian; 27.9% Hispanic/Latino; 15.7% Caucasian; 6.4% African American). Participants were screened for eligibility and excluded from participation if they were diagnosed with a cardiovascular disease, were regularly taking mood altering or cardiovascular altering medication, regularly smoked cigarettes, were not fluent in English, or did not have an iPhone. All participants were University of California, Irvine, students and consented to participate. Data collection took place from July 2018 through February 2019.

### Procedures

Participants underwent an approximately 90-minute laboratory session. All participant phones were confiscated at the beginning of the study under the pretext of measuring the external physical properties of the phone, which allowed the experimenter to later manipulate the phone conditions without arousing suspicion and ensure that all participants experienced similar circumstances of having their phone taken away. For participants randomly assigned to the *HRVB game* condition, the Happify app was installed on their phone and the experimenter guided them through the calibration settings of the Breather function while carefully concealing any indication that the purpose of using Breather was to reduce stress. Participants in the other conditions filled out surveys during this time. After participants completed a series of questionnaires and acclimated to the laboratory environment (approximately 25 minutes), the experimenter returned to the laboratory room and collected a baseline saliva sample. Participants were instructed in the passive drool technique of collecting their own saliva sample.

Participants then underwent a shortened version of the Trier Social Stress Task (TSST) [40,41] to induce psychological and physiological stress. The TSST consists of participants undergoing a public speaking task and arithmetic task in front of a panel of critically evaluative judges. The TSST has been shown to be a valid and reliable instrument for inducing physiological and psychological stress responses [42]. Immediately after the conclusion of the TSST, participants collected another saliva sample and self-reported their feelings of stress. For the next 5 minutes, participants were left alone in the room and interacted with their phone in a particular way depending on condition. Those in the *no phone* condition did not have their phone returned and were told to sit quietly for the next 5 minutes while the next portion of the study was prepared. Those in the *phone present* condition were given their phone but told “please do not use your phone for the remainder of the study.” Those in *HRVB game* condition were told to open the Happify app, navigate to Breather, and “follow the instructions on the app.” After the 5-minute phone manipulation period, the researcher returned to the room and instructed the participant to continue answering a series of questionnaires. Twenty minutes after completion of the TSST, a third saliva sample was collected. Forty minutes after the completion of the TSST, a fourth saliva sample was collected. At the conclusion of the study, the researcher and both judges debriefed the participant.

### Measures

#### Demographics and Covariates

Demographic information and potential covariates, including age, sex, ethnicity, socioeconomic status, perceived psychological stress, measures of daily phone use, time since waking, use of hormonal contraceptives, and caffeine intake, were collected via self-report.

#### Self-Reported Stress

Participants were asked to indicate, “How stressed do you feel right now?” on a visual analog scale from 1-100. This simple one-item scale has been shown to be valid and reliable for assessing perceptions of acute stress [43]. Self-reported stress was assessed at three time points (baseline, post-TSST, +20 minutes recovery).

#### Physiological Stress

Salivary cortisol and sAA were both collected to provide a broad assessment of the physiological stress response. Since these salivary biomarkers indicate activity of different physiological stress systems and have different secretion times, the inclusion of both gives us a more comprehensive understanding of stress effects. Salivary cortisol and sAA were collected using a passive drool technique with polypropylene cryovial salivettes at four time points. The first three samples were assayed for sAA, and the last three samples were assayed for cortisol to accommodate for the differing secretion times of each analyte (ie, an approximately 20-minute lag time for salivary cortisol secretion into saliva compared to immediate secretion of sAA) and ensure that the collection timing aligned to capture measures of baseline, post-TSST, and +20 minute recovery time points. Experimental sessions were conducted in the afternoon (between 1 PM to 6 PM) to account for the diurnal rhythm of sAA and cortisol.

Salivettes were stored at –80°C until batch analysis at the end of data collection at the laboratory of the Institute for Interdisciplinary Salivary Bioscience Research (University of California Irvine, Irvine, California). Before assaying, the samples were thawed for an hour to return them to room temperature. For cortisol, all samples were assayed in duplicate by using an expanded-range high-sensitivity salivary cortisol enzyme immunoassay kit (Salimetrics, LLC, State College, Pennsylvania). The assay range of sensitivity was 0.007 to 3.0 µg/dL, and the average intraassay coefficient of variation was 5.5%. For sAA, samples were tested in duplicate using a commercially available kinetic enzyme reaction assay kit (Salimetrics, LLC). The assay range of sensitivity was 0.4-400 U/mL, and the average intraassay coefficient of variation was 3.3.%.

### Analytic Strategy

All dependent variables (self-reported stress, sAA, and cortisol) were checked for skewness and kurtosis and transformed accordingly. No transformation was performed for values of self-reported stress. Values of sAA were moderately skewed, and a square root transformation was used to transform the values to approximate a normal distribution. Values of cortisol were moderately skewed, and a logarithmic transformation was used to transform the values to approximate a normal distribution. Outlying values above or below three SDs from the mean were removed. No outliers were removed for self-reported stress, three outliers (2.1%) were removed for sAA, and six outliers (2.8%) were removed for cortisol.

Models controlled for covariates that were significantly associated with the dependent variable. Sex, time since waking, and baseline cortisol were associated with cortisol recovery and were therefore controlled for in cortisol analyses. Baseline sAA was associated with sAA recovery and was therefore controlled for in sAA analyses. Baseline self-reported stress was associated with self-reported stress recovery and was therefore controlled for in self-reported stress analyses.

Independent sample *t* tests were used to conduct manipulation checks and ensure that exposure to the TSST reliably increased self-reported stress, sAA, and cortisol from baseline (time 1) to post-TSST stress (time 2). Repeated-measures mixed analysis of covariance was used to analyze the effect of condition on each dependent variable. Since the phone manipulation occurred after the TSST, analyses focused on differences in recovery and therefore used time (post-TSST stress at time 2 and +20 minute recovery at time 3) as the within-subject factor. Condition was included as a between-subject factor, and appropriate covariates were controlled for depending on the outcome of interest. Baseline values were controlled for to provide a more conservative and unbiased estimate of between-subject differences in composite recovery values [44]. Post-hoc comparisons were conducted to examine specific pairwise differences when a significant effect of condition was found.

## Results

### Manipulation Checks

Analysis of sAA from baseline (mean 85.31, SD 59.40) to post-TSST (mean 128.58, SD 88.53) revealed that participants displayed significant increases in sAA following the TSST (*t*_276_=–4.78, *P*<.001). In addition, analysis of cortisol from baseline (mean 0.21, SD 0.11) to post-TSST (mean 0.34, SD 0.24) showed that participants displayed significant increases in cortisol following the TSST, (*t*_271_=-5.99, *P*<.001). Finally, analysis of self-reported stress from baseline (mean 22.73, SD 21.48) to post-TSST (mean 47.31, SD 29.37) demonstrated that participants displayed significant increases in self-reported stress following the TSST (*t*_276_=–7.96, *P*<.001). These results indicate that TSST reliably increased psychological and physiological stress.

### Differences in Salivary Alpha Amylase Recovery Between Conditions

Between-subject comparisons indicated that there was a significant main effect of condition on sAA recovery (*F*_2,133_=3.78, *P*=.03; *no phone*: mean 10.078, SE 0.271; *phone present*: mean 10.007, SE 0.280; *HRVB game*: mean 9.132, SE 0.266). Post-hoc comparisons revealed that those in the *HRVB game* condition displayed significantly less sAA during recovery than those in the *no phone* condition (*t*_93_=2.48, *P*=.02) and the *phone present* condition (*t*_90_=2.26, *P*=.03). The *no phone* and *phone present* conditions did not differ (*t*_88_=–0.19, *P*=.85; Multimedia Appendix 2). Within-subject analyses revealed that there was no significant main effect of time for sAA recovery (*F*_1,133_=.003, *P*=.96) and no significant interaction between time and condition (*F*_2,133_=0.081, *P*=.92).

### Differences in Cortisol Recovery Between Conditions

Although the cortisol levels declined during recovery for all conditions, there was no main effect of condition on cortisol recovery (*F*_2,126_=1.19, *P*=.31).

### Differences in Self-Reported Stress Recovery Between Conditions

Although self-reported stress declined during recovery for all conditions, there was no main effect of condition on self-reported stress recovery (*F*_2,133_=1.42, *P*=.24).

## Discussion

### Principal Findings

In this study, we examined the effect of using or having a smartphone on psychological and physiological stress reduction during recovery. We found that those who used an HRVB training app exhibited the lowest levels of sAA during recovery. Specifically, those in the *HRVB game* condition released less sAA during recovery than those who had their phones present or had no phone at all. These results indicate that engaging in a brief 5-minute HRVB training session on a smartphone can effectively reduce stress-related sympathetic activity, as assessed by levels of sAA. Although the magnitude of the effect for the change in sAA was only small to medium (η^2^=0.05) [45], it was similar to previous studies [16,46]. The sAA findings are particularly important because high levels of sAA are associated with a range of deleterious health-related outcomes such as asthma, frequency of illness, and chronic fatigue [47-49]; therefore, lower levels of sAA are desirable from a health perspective. Our findings provide health-related information about the use of mHealth interventions on a smartphone. Since delayed physiological recovery can be predictive of risk for long-term health issues [50], we can infer that using a serious game such as Breather when recovering from a stressful experience may provide long-term health benefits.

Our study design did not allow us to conclusively determine the mechanisms responsible for the stress-buffering effect, but there are several possibilities for why Breather effectively aided in stress recovery. The most obvious explanation is that undergoing HRVB training increases parasympathetic activity, which is typically inversely related to sympathetic indicators such as sAA. Thus, the low levels of sAA for those in the *HRVB game* condition may be indicative of direct physiological alterations induced by the use of Breather. In addition, psychological factors may have played a role in explaining sAA recovery. The simple distraction induced by diverting cognitive attention away from ruminating thoughts about the stressor may have positively contributed to the effects. Furthermore, parasympathetic activity has been associated with increases in positive valence and low arousal emotions such as calmn [24], which suggests that feelings of calm may have also played a role in stress recovery. Finally, it is possible that the ability to monitor stress responses via the visual interface of Breather increased perceptions of control, which subsequently alleviated feelings of stress. This is due to the fact that acute stress is often induced by a perceived lack of control [51], and when that perceived control is increased, it can inhibit autonomic arousal [52]. It should be noted that no mechanisms can be determined for the lack of cortisol and self-reported stress, as we did not find significant effects on these measures. Future studies should further investigate the mechanisms for why HRVB delivered via a smartphone influences stress recovery.

Interestingly, those who had their phones present during stress recovery did not glean any additional stress-buffering benefits beyond those with no phone. Previous work has demonstrated that having a phone present, but not using it, leads to steep declines in sAA during physiological recovery from a stressor [16]. We failed to replicate this outcome. In the study by Hunter et al [16], participants had their phones with them while undergoing the stressor. In this study, participants only had their phones immediately after the study. This difference in timing implies that it may be helpful to have a phone present while experiencing a stressor, but it provides little to no benefit when present during recovery. In addition, a phone may serve as a “digital security blanket” in mildly stressful situations like social exclusion but may not exert similarly beneficial effects under more potent stressors such as the TSST.

### Limitations

There are several limitations that limit the generalizability of these results. First, our sample is not representative of the population at large. The majority of our participants were healthy young Asian women, all of whom were iPhone users and college educated. Since we drew our sample from a university population, our participants were likely wealthier, younger, more dependent on their phone, and more educated than the average person. Thus, these conclusions cannot be extrapolated to all populations.

In addition, the effectiveness of Breather for influencing sAA recovery compared to the other conditions may have been statistically limited by differences in baseline values. Those in the *HRVB game* condition had significantly lower levels of sAA at baseline. These differences may have been due to a methodological inconsistency, as the individuals in the *HRVB game* condition had a slightly different experience during the baseline period before undergoing the TSST; they spent approximately 2 minutes receiving training on the HRVB app. Per methodological recommendations, these baseline values were controlled for to provide a more conservative and unbiased estimate of between-subject differences in recovery [43]. Without the inclusion of baseline sAA as a covariate in the models, there would have been greater statistical differences between *HRVB game* use and the other conditions. Although this statistical decision does limit the magnitude of our sAA recovery findings, these differences in baseline raise an interesting point about the ways in which Breather influences reactions to a stressor. If, indeed, the brief training period reduced baseline sAA and sAA reactivity to the stressor, then using an HRVB serious game like Breather could possibly be an effective method for buffering stress reactivity as well as recovery and may be an advisable activity to engage in prior to a major stressor. Before any recommendations can be made, future studies should explore the optimal timing for HRVB implementation and determine whether it is most effective before or after a stressor.

Additionally, the effectiveness of Breather may have been hindered by the way in which the participant interacted with the app and understood the directions. During this training period, information about the purpose of using this app (eg, this activity makes you more relaxed and less stressed) was hidden from the participant in order to reduce demand characteristics and maintain internal validity across conditions. However, the success of biofeedback training hinges on the individuals’ perception that they are actively controlling their physiological functions in an effort to reduce stress [8]. Without this understanding about the purpose of the activity, the biofeedback exercise was likely less effective for the participants. In these ways, methodological constraints may have led to a more conservative effect of Breather compared to the other conditions.

Furthermore, user error issues that occurred within the app may have limited the effectiveness of Breather. The program requires the user’s finger to be placed very precisely on the light sensor to monitor heart rate change. It is sometimes difficult to maintain this position, and warnings pop up on the screen each time a finger is placed incorrectly. Based on participant feedback, these warnings made individuals feel as if they were performing poorly, which may have induced further stress rather than alleviate it. To investigate whether user error played a role, adherence to finger placement was assessed using metrics provided from the app’s database. Data showed that users had their fingers placed correctly for approximately 96% of the time; however, that still means that for 4% of the session, they were getting warnings telling them, “please place your finger on the sensor.” This may have been bothersome and unduly reduced the effectiveness of Breather, which is important to consider in future efficacy tests and real-life applications.

When considering a more comprehensive assessment of stress, conclusions from this study must be tempered by the lack of significant group differences for salivary cortisol and self-reported stress. Based on these discrepancies, we can only conclude that the HRVB game had a targeted effect on autonomic nervous system recovery as opposed to a general effect on all types of biological and psychological stress recovery. These inconsistencies in stress outcomes may be due to a variety of reasons. First, cortisol and sAA represent activity in different arms of the stress system and are not correlated at a 1:1 level [36,53]. Numerous studies have discovered significant sAA results, but not cortisol, during stress recovery [37,38]. The one study that examined both biomarkers in the context of phone usage only found significant sAA effects [16]. Our findings indicate that the HRVB training had a more robust impact on autonomic nervous system activity (indicated by sAA measures), which makes sense because HRVB training specifically targets fluctuations in cardiovascular activity that is intricately tied to autonomic activity [36]. In addition, the intervention period was short (about 5 minutes), which may not have been enough time to impact cortisol, often viewed as a chronic stress marker with a delayed release [53]. The discrepancy between self-reported and physiological stress is quite common in studies that assess both constructs [17,34,42] and one of the reasons many researchers argue for the importance of assessing both when contemplating health relevance of stress or psychological outcomes [54]. Additionally, there is substantial variation based on individual factors, such as demographics, in the association of subjective and objective measures of stress [55]. Furthermore, studies examining the convergence of self-report and physiological measures of stress have found that the assessments are highly correlated during the TSST, but not before or after [42]. Thus, it is not surprising that sAA was the only metric that yielded significant results. The significant sAA finding provides valuable information about how a HRVB training game via an mHealth app may aid in stress recovery; however, future studies should consider a wider range of health-related outcomes.

Finally, it should be acknowledged that the scope of this study did not allow us to conclusively determine whether using the HRVB app was more or less effective than performing other actions on one’s phone. Although past research is mixed on how phone use influences stress recovery [17,18], there is great potential for future researchers to explore how unstructured phone use (eg, listening to music, browsing social media) could impact physiological and psychological stress. Given the wide variety of potential ways in which people can use their phones, future studies should further investigate the effects of various types of phone interactions on stress recovery.

### Conclusions and Implications

Based on these results, one can conclude that completing HRVB training on an app such as Happify may be a practical and effective strategy for reducing acute physiological stress. It is often not feasible to use a smartphone to buffer stress while undergoing a stressor, but it is practical and ecologically valid to use a phone immediately after one has experienced a stressful experience. Our smartphones are conveniently with us at most times, and thus, we have this effective stress-reducing tool at our disposal anytime and anywhere we need it. To further examine how smartphones can aid in stress recovery, future research should investigate the mechanisms underlying how a gamified stress-reducing app may buffer stress and how it compares to other ways of using a phone. This will inform future interventions and provide recommendations for the development of other stress-buffering tools that can be delivered through smartphone apps.

Results such as these are beginning to change the narrative about the effect of smartphones on our well-being. Although it is important to recognize the deleterious effects of these devices on our lives, it may be even more critical to recognize the positive potential of smartphones and begin to develop and use technology in ways that augment well-being. Instead of simply hoping that individuals use technology in a beneficial manner, it is imperative that the hardware and software are designed in a way that facilitates positive behavior, thoughts, and interactions. Designing tools that take advantage of the technological affordances and ubiquity of smartphones to put stress-reducing tools in the palm of one’s hand is a promising strategy for finding ways for smartphones to maximize well-being.

## Appendix

Multimedia Appendix 1 A brief demo video of Breather on the Happify app.

Multimedia Appendix 2 Levels of salivary alpha amylase during recovery by experimental conditions.

## Abbreviations

HRV: heart rate variability
HRVB: heart rate variability biofeedback
mHealth: mobile health
mHealth: mobile health
sAA: salivary alpha amylase
TSST: Trier Social Stress Task
