# Supplementary figures and images for: Effect of Brief Biofeedback via a Smartphone App on Stress Recovery: Randomized Experimental Study
Source: JMIR Serious Games. 2019 Nov 26;7(4):e15974. doi: 10.2196/15974 (PMC6904898; doi:10.2196/15974)

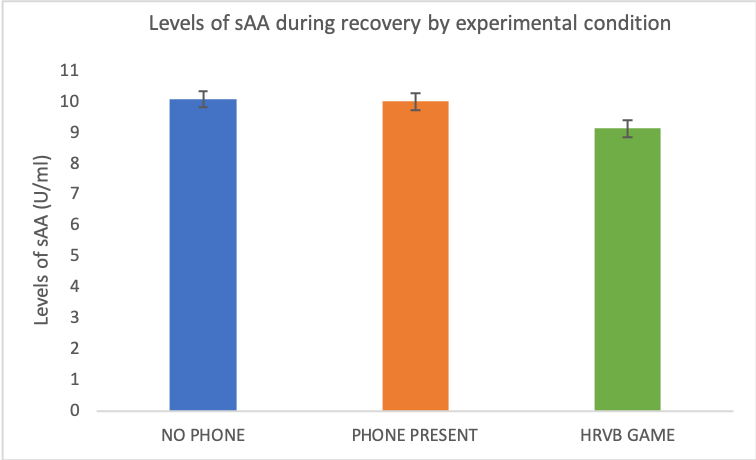

Supplement: Multimedia Appendix 2 [file games_v7i4e15974_app2.png]
